# Long-term exposure to air pollution and mammographic density in the Danish Diet, Cancer and Health cohort

**DOI:** 10.1186/s12940-015-0017-8

**Published:** 2015-04-01

**Authors:** Stephanie Huynh, My von Euler-Chelpin, Ole Raaschou-Nielsen, Ole Hertel, Anne Tjønneland, Elsebeth Lynge, Ilse Vejborg, Zorana J Andersen

**Affiliations:** Center for Epidemiology and Screening, Department of Public Health, University of Copenhagen, Copenhagen, Denmark; Department of Neuroscience, Smith College, Northampton, Massachusetts USA; Danish Center for Cancer Research, Danish Cancer Society, Copenhagen, Denmark; Department of Environmental Science, Aarhus University, Roskilde, Denmark; Diagnostic Imaging Centre, Copenhagen University Hospital, Copenhagen, Denmark

**Keywords:** Breast cancer, Mammographic density, Breast density, Air pollution, Nitrogen oxides, Traffic

## Abstract

**Background:**

Growing evidence suggests that air pollution may be a risk factor for breast cancer, but the biological mechanism remains unknown. High mammographic density (MD) is one of the strongest predictors and biomarkers of breast cancer risk, but it has yet to be linked to air pollution. We investigated the association between long-term exposure to traffic-related air pollution and MD in a prospective cohort of women 50 years and older.

**Methods:**

For the 4,769 women (3,930 postmenopausal) participants in the Danish Diet, Cancer and Health cohort (1993–1997) who attended mammographic screening in Copenhagen (1993–2001), we used MD assessed at the first screening after cohort entry. MD was defined as mixed/dense or fatty. Traffic-related air pollution at residence was assessed by modeled levels of nitrogen oxides (NO_x_) and nitrogen dioxide (NO_2_). The association between mean NO_x_ and NO_2_ levels since 1971 until cohort baseline (1993–97) and MD was analyzed using logistic regression, adjusting for confounders, and separately by menopause, smoking status, and obesity.

**Results:**

We found inverse, statistically borderline significant associations between long-term exposure to air pollution and having mixed/dense MD in our fully adjusted model (OR; 95% CI: 0.96; 0.93-1.01 per 20 μg/m^3^ of NO_x_ and 0.89; 0.80- 0.98 per 10 μg/m^3^ of NO_2_). There was no interaction with menopause, smoking, or obesity.

**Conclusion:**

Traffic-related air pollution exposure does not increase MD, indicating that if air pollution increases breast cancer risk, it is not via MD.

**Electronic supplementary material:**

The online version of this article (doi:10.1186/s12940-015-0017-8) contains supplementary material, which is available to authorized users.

## Introduction

Breast cancer is the most common type of cancer and one of the leading causes of death among women in the western world [1]. Still, only about one third of new cases are attributable to known risk factors, most of which are not easily modifiable for preventive purposes [2]. Breast cancer incidence is higher in more industrialized countries, as well in urban areas, suggesting, among other factors, a possible relevance of air pollution [3,4].

Epidemiological evidence is limited and mixed, but seems to increasingly suggest that air pollution, both from industrial sources [5,6] and traffic emissions [7-11] may increase breast cancer risk. The strongest evidence exist for the association of nitrogen dioxide (NO_2_), traffic related air pollutant, with breast cancer risk, provided by two Canadian case–control studies [8,11]. Crouse et al. reported a 25% increased risk of postmenopausal breast cancer for every 5 ppb increase in exposure to NO_2_ [8], whereas Hystad et al. found 26-32% increased risk of premenopausal breast cancer (and 7-10% for postmenopausal breast cancer) for every 10 ppb increase in exposure to NO_2_ [11].

Laboratory animal and toxicological data provide some evidence for a link between a number of carcinogens present in air pollution and breast cancer [1], most consistently for polycyclic aromatic hydrocarbons (PAHs), which have been found to cause mammary tumors in laboratory animals [12]. PAHs have both estrogenic and anti-estrogenic effects, and can cause oxidative stress [12]. Still, the exact biological mechanism behind a possible association between air pollution and breast cancer is uncertain.

One possible pathway could be via an intermediary such as mammographic density (MD), as suggested by a recent study reporting that women living in urban areas had greater MD than those living in rural areas [13]. MD is increasingly being used as a biomarker of breast cancer risk, as it is one of the strongest risk factors [14,15]. MD refers to the amount of radiologically dense breast consisting of epithelial or stromal tissue that appears light on a mammogram. Women with more than 75% density in the breast have a four to six times greater risk of breast cancer than women with little density, or fatty breasts [15,16]. Known determinants of MD include age at first birth, parity, age at menopause, hormone replacement therapy (HRT), and use of chemopreventive agents, such as tamoxifen, all of which are estrogen-related [17]. Estrogens, both endogenous and exogenous, have a proliferative effect on fibroglandular cells of the breast, increasing MD [18].

Environmental factors such as air pollution have yet to be assessed directly in relation to MD. Air pollution consists of a mix of carcinogens, including PAHs, which have been linked to breast cancer [1]. PAH derivatives can induce both estrogenic and anti-estrogenic activities [19], implying that it is plausible that exposure to air pollution can either increase or decrease MD.

The purpose of the present study was to investigate the association between long-term exposure to traffic-related air pollution and MD in the Danish Diet, Health and Cancer cohort.

## Materials and methods

The study population consists of 4,769 women above age 50 who participated in the Danish Diet, Cancer, and Health (DCH) cohort between 1993 and 1997 and subsequently attended the Copenhagen mammography screening program between 1993 and 2001.

### DCH cohort

Between 1993 and 1997, a total of 160,725 persons (72,729 women), 50 to 64 years of age, born in Denmark, living in Copenhagen or Aarhus (the two largest cities in Denmark), and with no record of cancer in the Danish Cancer registry, were invited to participate in the DCH cohort study. A total of 57,053 people, of whom 29,875 were women (37% of invited women and 7% of entire Danish female population in this age group), accepted the invitation and participated in the study, answering a comprehensive questionnaire on diet, health, education, occupation, lifestyle, and reproductive factors. A detailed description of the DCH cohort has been published previously [20]. Relevant Danish ethical committees and Danish Data Protection Agency have approved the study, and written informed consent was provided by all participants at recruitment.

### Copenhagen mammography register

The Copenhagen mammography screening program started in 1991 [21] and targeted approximately 40,000 women aged 50 to 69 years at the start of each biennial invitation round. We used data from the first five screening rounds between 1991 and 2001 [22]. Cases in which breast cancer was detected at the first screening were excluded from our final analytic data set, as these women lacked MD data.

### MD definition

One radiologist was in charge of the screening, which occurred at a single Copenhagen hospital. All screens were taken by the radiographers or x-ray nurses, and were evaluated independently by two radiologists, who did not meet the attending women. A two-view mammography, craniocaudal and oblique, was performed at the initial screening. MD was dichotomized into fatty breast, equivalent to Breast Imaging Reporting and Data System (BI-RADS) [23] density code 1 and part of code 2, and mixed/dense breast, equivalent to part of BI-RADS code 2, and BI-RADS code 3 or 4. Women with a negative screening test and fatty breasts were scheduled to have only an oblique view at their next screening, whereas women with a negative screening test and mixed/dense breasts were scheduled for another two-view mammography. MD was not coded for positive screening mammograms. The dichotomous outcome for MD has been successfully utilized in earlier studies, showing the expected associations with breast cancer risk [22,24]. Using the personal identification (CPR) number of the Danish Civil Registration System [25], we linked the Copenhagen mammography register to the DCH cohort. We used MD assessed at the first screening after the cohort baseline (1993–1997). MD did not change for women who participated in subsequent screens after cohort baseline until 2001.

### Exposure assessment

Air pollution was estimated using Danish AirGIS modeling system [26] used for estimating traffic-related air pollution with high temporal (an hour) and spatial (individual address) resolution. AirGIS (see: http://AirGIS.dmu.dk) calculates air pollution at a location as the sum of three contributors: (1) local air pollution from street traffic, calculated with the Operational Street Pollution Model (OSPM) from data on traffic (intensity and type), emission factors for each vehicle type, street and building geometry, and meteorology; (2) urban background, calculated from a simplified urban background (SUB) procedure that takes into account urban vehicle emission density, city dimensions (transport distance), and average building height; and (3) regional background, estimated from trends at rural monitoring stations and from national vehicle emissions. Input data for the AirGIS system come from various sources: a GIS-based national street and traffic database, including construction year and traffic data, a database on emission factors for the Danish car fleet, both dating back to 1960, a national geographical information system (GIS) database with building footprints supplemented with construction year and building height from the national building and dwelling register, national survey and cadastre data-bases, and a national terrain-evaluation model, provided the correct street geometry for a given year at a given address. With a geocoded address and a year, the starting point is specified in place and time, and the AirGIS system automatically generates street configuration data for the OSPM, including street orientation, street width, building heights in wind sectors, traffic intensity and type, and the other data required for the model. Air pollution is calculated in 2 m height at the facade of the building. The AirGIS system has been successfully validated and used in a number of studies [10].

AirGIS was used to calculate the outdoor concentration of NO_x_ and NO_2_ at current and historical residential addresses of DCH cohort members obtained from Danish Civil Registration System [25]. Only those cohort members with residential history information available for more than 80% of the time between 1971 and cohort baseline (97.4% of the total DCH cohort) were included. For these participants, missing values due to failed geocoding of an address (typically, one address out of several residential addresses between 1971 and cohort baseline was missing/failed geocoding, but maximum of 20% of the time between 1971 and cohort baseline) were substituted by the levels calculated for the previous known address or, when the first address was missing, for the subsequent address. We have thus obtained the complete dataset of annual mean NO_x_ and NO_2_ concentrations at the residential addresses (including moving) of cohort members from 1971 until cohort baseline (1993–1997). We used two definitions of long-term exposure to air pollution, in order to study the relevance of more recent or longer exposures to air pollution for MD, respectively: 1) baseline NO_x_ and NO_2_, defined as a 1-year mean levels at the residential address at the year at the cohort entry (1993/97); and 2) NO_x_ and NO_2_ since 1971, defined as a mean levels of annual concentrations NO_x_ and NO_2_ since 1971 until cohort entry (1993–1997), on average 25 year mean (22–26 year mean).

### Statistical methods

We used logistic regression to investigate the association of MD with four proxies of exposure to air pollution (baseline NO_x_ and NO_2_ and NO_x_ and NO_2_ since 1971) in separate models, in four steps: in a crude model adjusted for age (model 1); a model additionally adjusted for known determinants of MD: BMI (kg/m^2^), HRT use (ever/never), duration of HRT use (years), number of children, age at first birth, and history of benign breast disease (model 2); a model additionally adjusted for breast cancer risk factors: alcohol use (yes/no), alcohol intake (g/day), physical activity in leisure time (yes/no), and education (≤7 years/8-10 years/> 10 years) (model 3); and a model additionally adjusted for smoking status (current/ever/never), smoking duration (years), and smoking intensity (g/day), which is newly established risk factor for breast cancer, and exposure related to MD. Analyses were stratified by menopausal status and smoking status. Effect modification of an association of MD with four air pollution proxies by menopausal status and smoking status was analyzed by introducing interaction term into the model and tested by Wald test. Sensitivity an analysis was performed estimating association between air pollution and MD by quartiles of exposure to baseline NO_x_ and NO_2_ (presented in Additional file 1). Logistic procedure in R 3.1.1 was used to conduct the analyses. Results are presented as odds ratios (ORs) with 95% confidence intervals (95% CI) per 10 μg/m^3^ increase in NO_2_ and per 20 μg/m^3^ increase in NO_x_, which are standard units and utilize comparability with other air pollution studies with NO_2_ and NO_x_.

## Results

Among the 29,875 women of the DCH cohort, 7,507 between the ages of 50 and 69 were invited to screening by the Copenhagen mammographic screening program. We excluded 1,158 who did not participate in mammographic screening after cohort recruitment, 646 women who were positive at the first screen after recruitment, and 934 who lacked air pollution data, leaving a total of 4,769 participants in the final analytic data set. Mean time between cohort entry (1993–97) and mammographic screening was 1.1 years (90% were screened within 2 years of cohort baseline).

The mean age of the 4,769 women was 56.2 years, and the majority (56.7%) had mixed/dense breasts (Table 1). Mean age, BMI, and smoking duration were lower, whereas alcohol intake, smoking intensity, and mean age at first birth, were higher in women with mixed/dense breasts than in those with fatty breast, while there were no differences in HRT duration. Women with mixed/dense breasts were better educated and more physically active, and they were more likely be current smokers, premenopausal, and HRT users than women with fatty breasts.

**Table 1:** **Distribution of baseline characteristics for 4,769 women from Diet, Cancer, and Health cohort**

	**Total N = 4,769**	**Mammographic density**	
		**Mixed/dense N = 2,705**	**Fatty N = 2,064**
Baseline characteristics			
Mean (SD) age (years)	56.2 (4.4)	55.4 (4.3)	57.2 (4.4)
Menopausal, n (%)	3,930 (82.4)	2,147 (79.4)	1,783 (86.4)
Mean (SD) BMI (kg/m^2^)	25.9 (4.7)	24.6 (3.9)	27.6 (5.1)
Obese, n (%)	792 (16.6)	249 (9.2)	543 (26.3)
Short education (≤7 years), n (%)	1660 (34.8)	816 (30.2)	844 (40.9)
Medium education (8–10 years), n (%)	2355 (49.4)	1369 (50.6)	986 (47.8)
Long education (>10 years), n (%)	754 (15.8)	520 (19.2)	234 (11.3)
Alcohol use, n (%)	4,610 (96.7)	2623 (97.0 )	1987 (96.3)
Mean (SD) alcohol use in users (g/day)	13.9 (16.6)	14.8 (16.4)	12.8 (16.8)
Never smoked, n (%)	1814 (38.0)	1025 (37.9)	789 (38.2)
Previously smoked, n (%)	993 (20.8)	548 (20.3)	445 (21.6)
Current smoker, n (%)	1962 (41.1)	1132 (41.8)	830 (40.2)
Mean (SD) duration of smoking in ever smokers (years)	30.2 (11.8)	29.4 (11.6)	31.1 (11.9)
Mean (SD) smoking intensity in ever smokers (g/day)	10.0 (9.6)	10.2 (9.7)	9.7 (9.4)
Physically active, n (%)	2370 (49.7)	1384 (51.2)	986 (47.8)
Nulliparious, n (%)	662 (13.9)	455 (16.8)	207 (10.0)
History of benign breast tumor, n(%)	603 (12.6)	429 (15.9)	174 (8.4)
Ever used HRT	2295 (48.1)	1,398 (51.7)	897 (43.5)
Mean (SD) duration of HRT use in ever users (years)	5.6 (6.2)	5.6 (6.2)	5.6 (6.2)
Air pollution exposure at residential address			
Mean (SD) baseline NO_x_ (1-year mean 1993/97)	46.1 (42.4)	45.7 (41.5)	46.7 (43.5)
Mean (SD) NO_x_ since 1971 (ca. 25-year mean 1971-1993/97)	41.5 (30.7)	41.0 (30.0)	42.1 (31.6)
Mean (SD) baseline NO_2_ (1-year mean 1993/97)	23.0 (8.3)	23.0 (8.2)	23.1 (8.4)
Mean (SD) NO_2_ since 1971 (ca. 25-year mean 1971-1993/97)	21.2 (6.2)	21.1 (6.0)	21.3 (6.3)

SD - standard deviation, BMI - body mass index, HRT- hormone replacement therapy.

The mean levels of air pollution at residential addresses at baseline were 46.1 μg/m^3^ for NO_x_ and 23.0 μg/m^3^ for NO_2_, whereas the mean levels since 1971 were lower, 41.5 μg/m^3^ for NO_x_ and 21.2 μg/m^3^ for NO_2_ (Table 2). Women with mixed/dense breasts had lower levels of air pollution at residence than women with fatty breasts (Table 1).

**Table 2:** **Distribution of air pollution at residential address for 4,769 women from Diet, Cancer, and Health cohort**

	**Mean (SD)**	**Median (IQR)**	**25** ^**th**^ **-75** ^**th**^ **pct.**	**Min-Max**
*Air pollutants (μg/m* ^*3*^ *)*				
Baseline^a^ NO_x_	46.1 (42.4)	29.3 (26.7)	21.8-48.5	15.9-379.5
NO_x_ since 1971^b^	41.5 (30.7)	31.1 (23.8)	23.0-46.8	16.7-347.4
Baseline NO_2_	23.0 (8.3)	20.2 (7.9)	18.1-26.0	13.2-64.1
NO_2_ since 1971	21.2 (6.2)	19.7 (6.3)	17.4-23.7	13.2-59.6

SD - standard deviation; ^a^1-year mean 1993/97; ^b^ca. 25-year mean 1971-1993/97.

We found weak inverse, statistically non-significant associations of mixed/dense MD and four air pollution proxies in our age adjusted model (Model 1, Table 3). The associations became statistically borderline significant for all four air pollution proxies, after adjusting for HRT use, reproductive factors, and BMI (Model 2), and remained robust for further adjustment for education, alcohol intake and physical activity (Model 3), as well as for smoking (Model 4). Long-term exposure to air pollution, in our fully adjusted model (Model 4), was weakly inversely related to having mixed/dense breasts, with odds ratios (OR) (95% confidence intervals) of 0.97 (0.94-1.00) and 0.96 (0.93-1.01) per 20 μg/m^3^ increase in NO_x_ at baseline and NO_x_ since 1971, respectively, and 0.93 (0.86-1.00) and 0.89 (0.80-0.98) per 10 μg/m^3^ increase in NO_2_ at baseline and NO_2_ since 1971. Linear estimates are similar to ORs by quartiles of exposure to baseline NO_x_ and NO_2_ (see Additional file 1), showing none or weak inverse association, respectively.

**Table 3:** **Odds of having mixed/dense MD and long-term exposure to traffic-related air pollution (NO**_**2**_ **per 10 μg/m** ^**3**^ **, NO**_**x**_ **per 20 μg/m** ^**3**^ **) among 4,769 women**

	**Model 1** ^**a**^	**Model 2** ^**b**^	**Model 3** ^**c**^	**Model 4** ^**d**^
	**OR (95% CI)**	**OR (95% CI)**	**OR (95% CI)**	**OR (95% CI)**
*All women (n = 4,769)*				
Baseline NO_x_	0.99 (0.96-1.02)	0.97 (0.94-1.00)	0.97 (0.94-1.00)	0.97 (0.94-1.00)
NO_x_ since 1971	0.98 (0.94-1.02)	0.96 (0.92-1.00)	0.96 (0.92-1.00)	0.96 (0.93-1.01)
Baseline NO_2_	0.98 (0.92-1.06)	0.93 (0.86-1.00)	0.92 (0.85-1.00)	0.93 (0.86-1.00)
NO_2_ since 1971	0.95 (0.86-1.04)	0.89 (0.80-0.99)	0.89 (0.80-0.99)	0.89 (0.80-0.98)

OR - odds ratio; CI - confidence interval; ^a^adjusted for age; ^b^adjusted for Model 1 and BMI, HRT status, HRT duration, number of children; ^c^adjusted for Model 2 and alcohol use, alcohol intake (g/day), physical activity, education level; ^d^adjusted for Model 3 and smoking status, smoking duration, smoking intensity (g/day).

The associations observed for air pollution exposure since 1971 were marginally stronger compared to air pollution at baseline, for both NO_x_ and NO_2_. There was no statistically significant difference in the associations of any of the air pollution proxies and MD by menopausal status, nor with smoking status (Table 4).

**Table 4:** **Effect modification of the association** ^**a**^ **between MD and long-term exposure to traffic-related air pollution (NO**_**2**_ **per 10 μg/m** ^**3**^ **, NO**_**x**_ **per 20 μg/m** ^**3**^ **) by menopause and smoking status, among 4,769 women**

	**OR (95% CI)**	**OR (95% CI)**	***p-value***
	***Premenopausal n =*** **839**	***Postmenopausal n*** **= 3,930**	
Baseline NO_x_	0.95 (0.88-1.03)	0.98 (0.94-1.01)	0.62
NO_x_ since 1971	0.92 (0.83-1.03)	0.97 (0.92-1.01)	0.47
Baseline NO_2_	0.83 (0.67-1.01)	0.94 (0.87-1.02)	0.28
NO_2_ since 1971	0.77 (0.59-1.01)	0.91 (0.82-1.02)	0.27
	***Never smoker n = 1814***	**Ever smoker n = 2,955**	
Baseline NO_x_	0.96 (0.91-1.01)	0.98 (0.94-1.02)	0.66
NO_x_ since 1971	0.96 (0.89-1.03)	0.97 (0.92-1.02)	0.84
Baseline NO_2_	0.91 (0.80-1.03)	0.94 (0.85-1.03)	0.70
NO_2_ since 1971	0.90 (0.75-1.06)	0.90 (0.79-1.02)	0.99

BMI – body mass index; OR – odds ratio; CI – confidence interval; ^a^adjusted for age, BMI, HRT status, HRT duration, number of children; alcohol use, alcohol intake (g/day), physical activity, education level; smoking status.

## Discussion

We found no convincing association between exposure to traffic-related air pollution and MD. Associations were weak and inverse, and remained consistent with all four proxies of long-term exposure to air pollution, in both pre- and postmenopausal women, as well as by smoking status.

The evidence of an association between MD and air pollution is very limited, as this is the first study linking residential air pollution levels to MD. Results of our study are not consistent with findings by Perry et al., who has reported that women living in urban London had higher MD than women living in rural areas [13]. As exposure to air pollution is generally greater in urban areas, authors state that it is plausible that air pollution increases MD, thereby also raising breast cancer risk. Perry et al. furthermore claims that traffic emissions particles have significant estrogenic activity [13,27,28], thus supporting his conclusion that air pollution may increase breast cancer risk through a mechanism that acts via MD. However, the study by Perry et al. has several limitations precluding a conclusion on the association between air pollution and MD. Perry et al. did not have data on air pollution, only on urban status of women’s residences and their MD. Furthermore, Perry et al. did not adjust for any risk factors for MD or breast cancer, possibly leading to biased results due to possible confounding by socioeconomic status, age, reproductive factors, HRT status, menopausal status, etc. In conclusion, in the first prospective cohort study on the topic, we found that exposure to traffic-related air pollution is not positively, but weakly inversely associated with MD, and thus likely does not increase breast cancer risk via MD. This novel finding needs to be reproduced.

Our results can be compared to those of studies investigating the association of MD with smoking, an exposure related to air pollution. Many of the carcinogens found in tobacco smoke are also found in air pollution, and both have similar toxicological properties and biological mechanisms of damage to the human body [29]. Thus, a similar direction and magnitude of the association between MD with both smoking and air pollution is expected. Although the relationship between smoking and breast cancer has been a source of debate, recent studies, including one that also used the DCH cohort seen in our study [30], provide consistent and strong evidence of a positive association [30-33]. The evidence on association between smoking and MD is mixed, but points toward either a weak inverse or no association, in agreement with our findings on air pollution and MD. Three studies failed to detect association between smoking and MD [33-35], but the majority found an inverse association [36-40]. This is consistent with an antiestrogenic effect of tobacco smoke, which has been illustrated earlier [41]. Results of our study and current evidence thus point to opposing effects of air pollution on breast cancer and MD, resembling the effects of tobacco smoke: increasing breast cancer risk, while having no effect, or possibly weakly decreasing MD. Therefore, we tentatively conclude that if air pollution increases breast cancer risk, it is likely via a pathway that is independent of MD.

Strengths of our study include a large prospective cohort, with well-defined and validated information on MD [24], as well as all relevant MD and breast cancer risk factors, which were prospectively collected, therefore limiting the possibility of recall, or information bias. We furthermore benefited from the state-of-the art information on exposure to air pollution at residence with high spatial (address-specific) and temporal (annual mean) resolution, assessed since 1971 years, capturing chronic exposure to air pollution. The air pollution models used to assess NO_x_ and NO_2_ levels have been successfully validated [26] and applied in a number of previous epidemiological studies [10]. Furthermore, this is the first study to investigate association between and air pollution to MD, a novel biomarker of breast cancer risk.

The main limitation of this study is the possible exposure misclassification of modeled air pollution concentrations at residence, which are only proxies of personal exposure. We did not have data on other pollutants such as particulate matter (PM) or carbon dioxide (CO), and used NO_x_ and NO_2_ concentrations as our primary exposure variables because they correlate strongly with other traffic-related pollutants present in Danish streets, such as the ultrafine particles emitted from diesel engines [10]. Furthermore, exclusion of cohort members due to missing data on air pollution exposure, due to missing address or address geocode, is a limitation. This is however not likely to bias our results, as missing addresses are not systematically related to air pollution levels, but due to address register incompleteness (missing street code or house number, typically for early addresses). Exposure misclassification could also arise from the lack information regarding indoor air pollution exposures, use of air purifiers and air conditioners, work address and related exposure to air pollution at work, working time, transportation habits, and outdoor activity patterns. Exposure misclassification from various sources would bias our results towards zero. Air pollution levels are relatively low in Copenhagen, and these findings need to be reproduced in sites with higher air pollution levels. In addition, excluding studies looking at MD in relation to smoking, there is currently no other evidence from preceding research that supports the validity of our findings, and more studies will be needed to confirm or refute this observed weak inverse association between air pollution and MD.

### Consent

Written informed consent was obtained from the patient for the publication of this report and any accompanying images.

## Additional file

Additional file 1:
**Sensitivity Analyses.**
