# Supplementary material for: Long-term exposure to air pollution and mammographic density in the Danish Diet, Cancer and Health cohort
Source: Environ Health. 2015 Apr 1;14:31. doi: 10.1186/s12940-015-0017-8 (PMC4392475; doi:10.1186/s12940-015-0017-8)
Supplement: Additional file 1: — Sensitivity Analyses. [file 12940_2015_17_MOESM1_ESM.docx]

**Additional File 1**

**Sensitivity Analyses**

**Table S3.** Odds^a^ of having mixed/dense MD and long-term exposure to traffic-related air

pollution at baseline residential address among 4,769 women.

| **Air Pollutant by quartiles of exposure** | **OR (95%CI)** |
| --- | --- |
| Baseline NO_x_ < 21.8 | 1.00 |
| Baseline NO_x_ ≥ 21.8 & < 29.3 | 1.02 (0.84-1.23) |
| Baseline NO_x_ ≥ 29.3 & < 48.5 | 0.87 (0.72-1.06) |
| Baseline NO_x_ ≥ 48.5 | 0.89 (0.73-1.07) |
| Baseline NO_2_ < 18.1 | 1.00 |
| Baseline NO_2_ ≥ 18.1 & < 20.2 | 0.97 (0.81-1.15) |
| Baseline NO_2_ ≥ 20.2 & < 26.0 | 0.83 (0.69-0.99) |
| Baseline NO_2_ ≥ 26.0 | 0.90 (0.75-1.08) |

OR - odds ratio; CI - confidence interval; Baseline - 1-year mean 1993/97; ^a^Fully adjusted (Model 4 in Table 3) for age, BMI, HRT status, HRT duration, number of children, alcohol use, alcohol intake (g/day), physical activity, education level, smoking status, smoking duration, smoking intensity (g/day).
